# Low-dose amitriptyline for irritable bowel syndrome: a qualitative study of patients’ and GPs’ views and experiences

**DOI:** 10.3399/BJGP.2024.0303

**Published:** 2025-03-11

**Authors:** Emma J Teasdale, Hazel A Everitt, Sarah L Alderson, Alexander C Ford, James Hanney, Matthew Chaddock, Emmajane Williamson, Heather Cook, Amanda J Farrin, Catherine Fernandez, Elspeth A Guthrie, Suzanne Hartley, Amy Herbert, Daniel Howdon, Delia Muir, Sonia Newman, Pei Loo Ow, Matthew J Ridd, Christopher M Taylor, Ruth Thornton, Alexandra Wright-Hughes, Felicity L Bishop

**Affiliations:** Centre for Clinical and Community Applications of Health Psychology, School of Psychology, University of Southampton, Southampton.; Primary Care Research Centre, Faculty of Medicine, University of Southampton, Southampton.; Leeds Institute of Health Sciences, School of Medicine, University of Leeds, Leeds.; Leeds Gastroenterology Institute, St James’s University Hospital, Leeds; Leeds Institute of Medical Research at St James’s, University of Leeds, Leeds.; Centre for Clinical and Community Applications of Health Psychology, School of Psychology, University of Southampton, Southampton.; Let’s Cure IBS (IBS support group), Leeds.; Let’s Cure IBS (IBS support group), Leeds.; Exeter Clinical Trials Unit, University of Exeter, Exeter.; Clinical Trial Research Unit, Leeds Institute of Clinical Trials Research, School of Medicine, University of Leeds, Leeds.; Clinical Trial Research Unit, Leeds Institute of Clinical Trials Research, School of Medicine, University of Leeds, Leeds.; Leeds Institute of Health Sciences, School of Medicine, University of Leeds, Leeds.; Clinical Trial Research Unit, Leeds Institute of Clinical Trials Research, School of Medicine, University of Leeds, Leeds.; Population Health Sciences, Bristol Medical School, University of Bristol, Bristol.; Leeds Institute of Health Sciences, School of Medicine, University of Leeds, Leeds.; Clinical Trial Research Unit, Leeds Institute of Clinical Trials Research, School of Medicine, University of Leeds, Leeds.; Primary Care Research Centre, Faculty of Medicine, University of Southampton, Southampton.; Clinical Trial Research Unit, Leeds Institute of Clinical Trials Research, School of Medicine, University of Leeds, Leeds.; Population Health Sciences, Bristol Medical School, University of Bristol, Bristol.; Clinical Trial Research Unit, Leeds Institute of Clinical Trials Research, School of Medicine, University of Leeds, Leeds.; Primary Care Research Centre, Faculty of Medicine, University of Southampton, Southampton.; Clinical Trial Research Unit, Leeds Institute of Clinical Trials Research, School of Medicine, University of Leeds, Leeds.; Centre for Clinical and Community Applications of Health Psychology, School of Psychology, University of Southampton, Southampton.

**Keywords:** amitriptyline, irritable bowel syndrome, patient experience, primary care, qualitative research

## Abstract

**Background:**

Irritable bowel syndrome (IBS) can cause troublesome symptoms, which impact patients’ quality of life and incur considerable health service resource use. Guidelines suggest low-dose amitriptyline for IBS as second-line treatment, but this is rarely prescribed in primary care.

**Aim:**

To explore patients’ and GPs’ views and experiences of using low-dose amitriptyline for IBS.

**Design and setting:**

Qualitative interview study with patients and GPs in England, nested within the ATLANTIS trial of low-dose amitriptyline versus placebo (ISRCTN48075063).

**Method:**

Semi-structured telephone interviews were conducted with 42 patients at 6 months post-randomisation, with 19 patients again at 12 months post-randomisation, and with 16 GPs between April 2020 and March 2022. Reflexive thematic analysis was used to analyse patient and GP data separately, then together, to identify unique and cross-cutting themes.

**Results:**

We found concerns about amitriptyline being an antidepressant, medicalising IBS, and side effects. Perceived benefits included the low and flexible dose, ease of treatment, and familiarity of amitriptyline and its potential to offer benefits beyond IBS symptom relief. These concerns and perceived benefits were expressed in the context of desire for a novel approach to IBS: GPs were keen to offer more options for IBS and patients sought a cure for their symptoms.

**Conclusion:**

Patients and GPs felt that the potential benefits of trying low-dose amitriptyline for IBS outweighed their concerns. When offering low-dose amitriptyline for IBS, GPs could address patient concerns about taking an antidepressant for IBS, highlighting the low and flexible dosage, and other potential benefits of amitriptyline such as improved sleep.

## Introduction

Irritable bowel syndrome (IBS) is a common, chronic, functional bowel disorder that is characterised by abdominal pain and altered bowel habit. Symptoms range from mild to severe, and can often be recurring and erratic.^1^ IBS has a substantial impact on patients’ quality of life and social functioning, which incurs considerable health service resource use.^2^ The prevalence of IBS in the community is around 10%,^3^ and IBS accounts for more than 3% of all consultations in primary care,^4^ costing the UK health service over £200 million/year.^5^

Current treatment of IBS in primary care includes dietary and lifestyle advice, and the use of laxatives, and antimotility and antispasmodic drugs.^6^ If these treatments are ineffective, the National Institute for Health and Care Excellence (NICE) guidance recommends that GPs consider prescribing low-dose tricyclic antidepressants, such as amitriptyline, as second-line treatment for IBS.^7^^,^^8^ There is evidence to suggest that low-dose amitriptyline might improve IBS symptoms as a result of its pain-modifying properties^9^^–^12 and influence on gastrointestinal motility,^13^^,^^14^ and so acts as a neuromodulator at low dose.

Previous qualitative research has explored the impact of IBS on patients’ physical health, psychological and social wellbeing, daily activities, and experiences of treatment seeking.^15^^–^20 The diagnostic process is seen as confusing, and patients are frequently frustrated by prolonged searches for effective treatments, which can reduce their trust in doctors.^16^^,^^17^ More support from healthcare professionals and initiatives to combat the societal stigma of IBS are needed to help people cope with the disruption to their lives caused by painful and uncomfortable physical symptoms, loss of social and employment activity, and psychological distress.^21^

**Table:** How this fits in

Low-dose amitriptyline is recommended in National Institute for Health and Care Excellence guidance for patients with irritable bowel syndrome (IBS) if first-line treatments are ineffective, but it is infrequently prescribed in primary care. Greater insight into the factors affecting prescribing and uptake of low-dose amitriptyline for IBS in primary care may improve outcomes for people with IBS. This study found that patients and GPs felt that the potential benefits of trying low-dose amitriptyline for IBS outweighed their concerns about taking an antidepressant for IBS, and highlights how concerns can be addressed.

The ATLANTIS (Amitriptyline at Low-dose and Titrated for Irritable Bowel Syndrome as Second-Line Treatment in primary care) trial evaluated the clinical and cost-effectiveness of low-dose amitriptyline versus placebo as a second-line treatment for IBS in primary care.^22^^,^^23^ Nested within the main trial, this qualitative study explored patients’ and GPs’ experiences of trial treatments and processes, aiming to support the interpretation of trial outcomes and inform future efforts to promote wider use of amitriptyline for IBS, if appropriate. This article focuses on patients’ and GPs’ views and experiences of using low-dose amitriptyline for IBS in primary care. The analysis was conducted before the trial results were available. The ATLANTIS trial results have since been published^23^ and indicate that low-dose amitriptyline for IBS is effective and safe.

## Method

### Design

This was a nested qualitative study comprising semi-structured telephone interviews with a sub-sample of patients and GPs from West Yorkshire, West of England, and Southern England in the ATLANTIS trial. The qualitative team comprised a female research fellow with experience of conducting qualitative research about IBS, a female health psychologist, two female GPs, two students (one male and one female), and two patient and public involvement (PPI) contributors (one male and one female). Four core members of the research team held monthly meetings to review study progress, review interview data, and collaborate on data analysis. Bi-monthly meetings were held with PPI members to seek their input in these activities. This study is reported in accordance with the Standards for Reporting Qualitative Research.^24^

### Participants and recruitment

#### Patients

Adults aged ≥ 18 years with a GP diagnosis of IBS and ongoing troublesome symptoms were invited via GP practice mailouts to participate in the trial. Between October 2019 and April 2022, 463 patients were recruited. During the trial consent procedure, patients optionally consented to be contacted about the qualitative study. After 6 months in the trial, the ATLANTIS trial team based at Leeds Clinical Trials Research Unit sent consenting patients a qualitative study invitation pack comprising an invitation letter, participant information sheet, and qualitative interview consent form. Interested patients emailed completed consent forms to the qualitative team, who arranged a mutually convenient time for interview. One hundred and forty invitations were sent between April 2020 and March 2022. Patient interviewees were also invited to a follow-up interview at 12 months post-randomisation. Two researchers conducted 61 telephone interviews (42 initial and 19 follow-ups). Of the 42 participants, only 29 could be contacted for a follow-up interview. Because of recruitment delays caused by the COVID-19 pandemic, the remaining 13 participants had not reached their 12-month timepoint.

#### GPs

Forty-two of the 55 ATLANTIS general practices were available to be contacted during the qualitative study timeframe. One researcher sent the GP information sheet and consent form to these practices following their patient recruitment period (approximately 5 months after they completed their mailout).^22^ GPs interested in taking part emailed a completed consent form to the qualitative team. One researcher conducted 15 interviews, and another researcher conducted one interview, between October 2020 and March 2022.

#### Sampling

We used convenience sampling, rather than sampling for variation as planned, because of delays to ATLANTIS caused by the COVID-19 pandemic. All patients and GPs who expressed interest, consented, and responded to the qualitative researcher’s request to arrange an interview were included. Nevertheless, interviewees varied in their personal and professional characteristics (Tables 1 and 2). In terms of treatment arm, 55% (*n* = 23) of patient interviewees were allocated to the amitriptyline arm and 45% (*n* = 19) were allocated to the placebo arm. Patients and interviewers remained blinded to treatment allocation during interviews (except seven patients who were unblinded, as per trial procedures, before their 12-month interview).

**Table 1.** Baseline characteristics of interviewees at 6 and 12 months, and whole trial sample

**Baseline characteristics**	**6 months (*n* = 42)**		**12 months ( *n* = 19)**		**All trial participants (*n* = 463)**	

	***n***	**%**	***n***	**%**	***n***	**%**
**Sex**						
Female	30	71%	15	79%	315	68%
Male	12	29%	4	21%	148	32%

**Age**						
Median age (years)	54	—	54	—	49	—
Range (years)	21–83	—	25–83	—	19–87	—

**Recruitment hub**						
Wessex	16	38%	6	32%	192	41%
West Yorkshire	9	22%	6	32%	87	19%
West of England	17	40%	7	36%	184	40%

**Education level**						
No formal	1	2%	1	5%	31	7%
GCSE	7	17%	3	16%	122	26%
A level	11	26%	5	26%	108	23%
Degree	9	22%	4	22%	110	24%
Postgraduate	13	31%	5	26%	78	17%
Diploma	1	2%	1	5%	6	1%
Other	0	0%	0	0%	6	1%
Missing					2	1%

**Ethnicity**						
White	41	98%	18	95%	451	97%
Asian	1	2%	1	5%	4	1%
Black	0	0%	0	0%	1	0%
Other ethnic groups	0	0%	0	0%	2	1%
Mixed	0	0%	0	0%	3	1%
Prefer not to say	0	0%	0	0%	1	0%
Missing					1	0%

**IBS-SSS at baseline**						
Mild	9	22%	4	21%	63	14%
Moderate	19	45%	9	47%	201	44%
Severe	14	33%	6	32%	191	42%

**Trial arm**						
Amitriptyline	23	55%	9	47%	232	50.1%
Placebo	19	45%	10	53%	231	49.9%

*IBS-SSS = irritable bowel syndrome symptom severity score*

**Table 2.** Demographic data for GP interviewees (*n* = 16)

**Variable**	***n***	**%**
**Sex**		
Female	8	50%
Male	8	50%

**Age, years**		
Median age	45	—
Range	34–60	—

**Recruitment hub**		
Southampton	7	44%
West Yorkshire	0	0%
West of England	9	56%

**Ethnicity**		
White	12	75%
Asian	3	19%
Mixed	1	6%

**Employment status**		
Part-time	11	69%
Full-time	5	31%

**Years worked as GP**		
Median	18	—
Range	3–30	—

### Data collection

Supplementing previous written informed consent, interviewers obtained verbal consent before starting each interview. Two topic guides comprising open-ended questions were developed by the qualitative team, drawing on existing literature and input from a patient collaborator. Interviewers used topic guides flexibly to remain open to exploring interviewees’ individual experiences and perspectives in depth, allowing novel and unanticipated insights. The patient topic guide (see Supplementary Box S1 for details) was informed by the common-sense model of illness perception,^25^ which provides a framework for understanding how people experience treatments and make treatment decisions within the context of chronic illness; this has proved relevant in previous qualitative work on IBS.^19^ The GP topic guide (see Supplementary Box S2 for details) was informed by key domains from normalisation process theory,^26^ which provides a framework for identifying factors and processes likely to hinder or enable widespread implementation of new practices.

Interviews were audio-recorded using a digital recorder (except for one interview that was audio-recorded using Microsoft Teams). Field notes were taken to capture the interviewers’ impressions and reflections, and any aspects not captured by the audio-recorder. GP interviews lasted 18–45 minutes (mean 27 minutes). Patient interviews lasted 17–65 minutes (mean 40 minutes) at 6 months, and 15–60 minutes (mean 29 minutes) at 12 months. On finishing each interview, interviewees were thanked and debriefed. They were offered a copy of their transcript and study findings, when available. Data collection stopped on reaching thematic saturation (that is, when themes were rich, well developed, and understood) and a rigorous, credible analysis in relation to our aims had been achieved.

### Data analysis

Before analysis, interviews were transcribed verbatim by a professional transcription company and any identifiable data removed. Reflexive thematic analysis^27^^,^^28^ was used to explore the data (Box 1). Data collection and initial analyses proceeded iteratively (that is, coding started after the first few interviews and informed subsequent interviews).

**Box 1.** Detailed description of data analysis method

**Six phases of reflexive thematic analysis**	**Description of tasks undertaken**
**Phase 1: Data familiarisation**	One researcher repeatedly read through all the transcripts and listened back to the audio- recordings. Three other core members of the qualitative team read at least four transcripts to familiarise themselves with the data
**Phase 2: Generating initialcodes**	One researcher conducted line- by- line coding. Patient 6- month and 12- month interviews, and GP interviews, were all coded separately. Codes were derived inductively from the data and grouped together to produce separate initial coding frames for the three interview datasets. Initial codes were reviewed and discussed with the core qualitative team and PPI representatives
**Phase 3: Searching for themes**	After coding all the transcripts, one researcher sorted codes into potential themes by recognising meaningful repeated patterns and identifying key concepts in the data. To enhance the quality and credibility of the analysis, detailed coding manuals were produced containing names and descriptions of potential themes and sub-themes, along with example quotes. Two coding manuals were produced — one for GP data and one for 6- and 12- month patient data. These were reviewed with the core team at monthly meetings
**Phase 4: Reviewing themes**	Potential themes and sub- themes were discussed with, and iteratively developed by, members of the qualitative team and PPI members to offer diverse interpretations of the data at regular meetings.^29^^,^^30^ Reviewing themes for fit with coded extracts and entire dataset involved reviewing the original data for relevant incidents for each potential theme and expanding, merging, or generating new themes. A negative case analysis was carried out, which explicitly searched for ideas in the data that were potentially inconsistent with our interpretations. This allowed us to identify and reflect on important, but rare, views and the limits of the analysis
**Phase 5: Defining and naming themes**	Matrices and diagramming were used to compare the themes’ similarities and differences between the 6- month and 12- month patient interviews and between the patient and GP interviews. Themes were reviewed and refined to form broader cross-cutting themes, producing an overarching narrative that drew on the patient 6- month, patient 12- month, and GP interviews
**Phase 6: Reporting**	Quotes were reviewed and discussed by the qualitative team to provide compelling examples for theme and sub- themes and to write up our findings

*PPI = patient and public involvement.*

Although analysis was primarily inductive (data-driven), the common-sense model of illness perception^25^ and normalisation process theory^26^ were consulted during analysis to aid our interpretation. NVivo (version 12) was used to manage data, implement and record coding, and to perform thematic comparisons described above.

## Results

**Figure 1.**
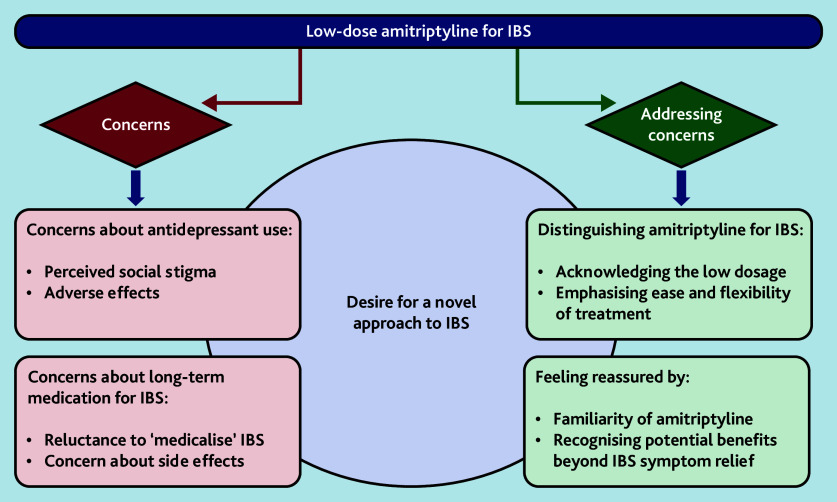
Key themes: concerns and perceived benefits to using low-dose amitriptyline for IBS. IBS = irritable bowel syndrome.

**Figure 2.**
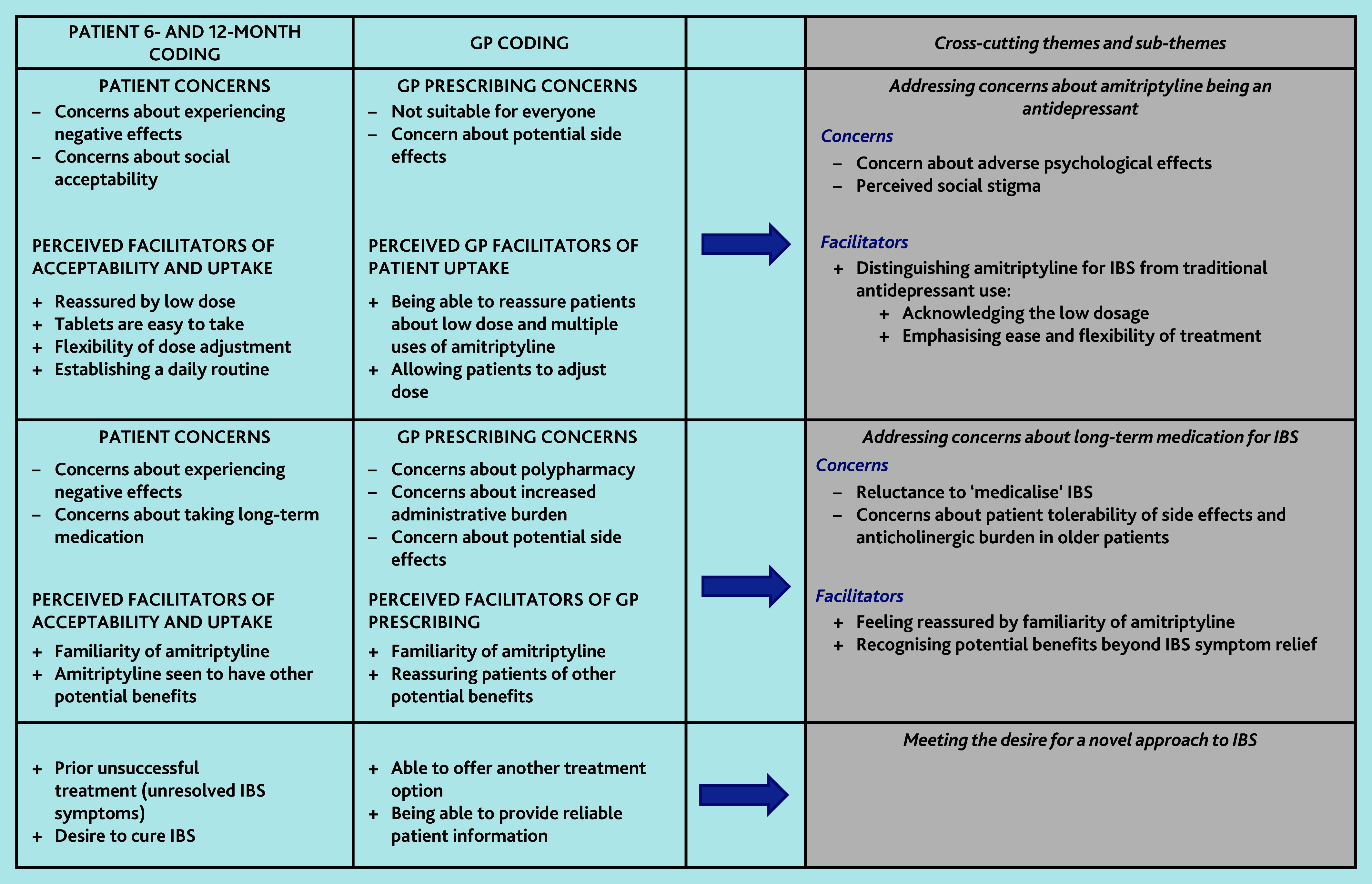
Thematic map IBS = irritable bowel syndrome.

We identified concerns and perceived benefits to using low-dose amitriptyline for IBS from GPs’ and patients’ perspectives, as well as an overarching theme that explained why patients and GPs in this trial were willing to try low-dose amitriptyline despite some concerns (Figure 1). Three cross-cutting themes encompassed both concerns and potential benefits: addressing concerns about amitriptyline being an antidepressant; addressing concerns about long-term medication for IBS; and meeting the desire for a novel approach to IBS (Figure 2). We explore these themes below and present selected illustrative anonymised quotes.

### Addressing concerns about amitriptyline being an antidepressant

An overriding concern raised by both patients and GPs was about amitriptyline being an antidepressant.

#### Concern about adverse psychological effects

Patients expressed concerns about experiencing adverse psychological effects with thoughts such as that taking amitriptyline might *‘alter my mind’* (P2), *‘mess with my head’* (P4), or *‘zombify me’* (P14). Another predominant concern for patients was that they might experience stigma for taking an antidepressant, as amitriptyline is not commonly viewed as a treatment for IBS:

*‘The worst thing about it is that it can be used as an antidepressant, and it’s almost like, if somebody saw that on your prescription, they wouldn’t think, “oh, she’s taking that because she has IBS”; they would think, “oh, she must have stress or depression”, and there is still a stigma about that. I think that’s probably the biggest negative, is that kind of stigma.’*

(P22, female patient aged 55–64 years with mild IBS, 12-month interview)

#### Perceived social stigma

The perceived social stigma associated with taking an antidepressant was also recognised by GP interviewees. They reflected on the potential for patient reticence towards taking an antidepressant for a *‘functional physical health problem’* (GP3), especially in patients with mild symptoms and how this could affect their prescribing decisions:

*‘In patients potentially with mild symptoms, they may be reluctant to use a medication that’s labelled an antidepressant for their physical health problem, and there are quite a number of patients who are reluctant to use antidepressants even when they have depression.’*

(GP2, male GP aged 35–44 years with 15–20 years’ experience)

#### Distinguishing amitriptyline for IBS from traditional antidepressant use

Distinguishing between the use of amitriptyline for IBS and its traditional use as an antidepressant may address such concerns and thus potentially facilitate uptake. Acknowledging the low dosage of amitriptyline for IBS treatment was important to patients as they felt this reduced the likelihood of adverse effects and helped them distinguish it from being *‘just a treatment for depression’* (P23):

*‘I personally don’t have an issue with it and knowing how the dosages work in terms of treating mental illness compared to those levels that were being suggested for this.’*

(P38, male patient aged 45–54 years with moderate IBS, 6-month interview)

Similarly, GPs felt that it was important to reassure patients that amitriptyline, when prescribed at a much lower dose than when used for depression, is used for other conditions for other effects, such as pain reduction. They also felt that emphasising to patients that amitriptyline is prescribed for their ‘physical symptoms’ helps to validate patients’ experience of symptoms and to address potential misunderstandings, where patients might think amitriptyline is being prescribed *‘because you think it’s all in the mind’* (GP13):

*‘I think often patients can be worried that amitriptyline is* — *they’ve heard of it as an antidepressant and they’re worried about it from* [that] *point of view, but if you explain to them that we’re not using it for those properties and it’s a much lower dose and it’s very safe, et cetera, it actually is quite well accepted.’*

(GP6, female GP aged 45–54 years with 15–20 years’ experience)

Emphasising the ease and flexibility of treatment was also viewed as important. Patients particularly appreciated the small size of the tablets making them *‘easy to swallow’* (P6) and only having to take tablets once a day, making it easy to fit into existing daily routines:

*‘It’s just become part of the norm now. It’s in the kitchen in the container and come six o’clock I just take the three pills and my phone goes off to remind me. I’ve had no problems taking it or anything like that. They’re the smallest tablets in the world!’*

(P10, male patient aged 55–64 years with mild IBS, 6-month interview)

Another element of perceived ease of treatment was individualisation of the dose (being able to adjust the dose from one 10 mg tablet [10 mg] up to three 10 mg tablets [30 mg] and down again). Although some patients were uneasy about adjusting the dose themselves, and felt they would prefer any dose adjustment to be a medical judgement*,* most patients felt empowered and appreciated having the flexibility to adjust the dose according to their needs and at their own pace — *‘having control over it and going with what my body felt like’* (P17). They found the trial dose titration instructions and flowchart straightforward and helpful. GPs were generally positive about patients having the ability to individualise their dose to suit their needs. They reflected that patient self-titration was a good idea, one which could empower patients as well as reduce appointment time — *‘otherwise that’s three appointments just to get them to thirty milligrams’* (GP3).

### Addressing concerns about long-term medication for IBS

#### Reluctance to ‘medicalise’ IBS

Broader concerns were expressed, particularly by GPs, about the consequences of prescribing a long-term ‘drug’ such as amitriptyline for IBS. GPs predominantly worried that prescribing amitriptyline could ‘medicalise’ IBS and increase the administrative burden of repeat prescriptions and review consultations, and they highlighted potential contraindications to its prescription and polypharmacy issues:

*‘My general view is that if you can manage with lifestyle changes and not drugs, that is a better thing to do. I think it’s an established well-known drug and if it works it would be cheap and if it’s effective that is good. It’s just what happens longer term, you know? Whether it is something, it would be interesting to know whether you can have it for a bit and then withdraw it, for example. Rather than it becoming another long-term drug to add to the burden of polypharmacy that we have!’*

(GP1, female GP aged 55–64 years with over 20 years’ experience)

A reluctance to ‘medicalise’ IBS was also evident in the patient data. Patients expressed concerns about drug side effects or dependency — *‘Initially I was a little bit worried. I didn’t want any side effects’* (P12), *‘I was worried that I might get addicted to it in some way’* (P6) — but this seemed to have been tempered by the sense that, if they experienced any adverse effects, it would be ‘known’ side effects and, as such, manageable. This may be because patients had all signed up for the ATLANTIS trial and so had been provided with detailed information about possible side effects:

*‘I suppose with any low-dose medication that can be used for depression, anxiety, and whatnot, I did think weight gain might be part of it, but again I thought if it helps with my stomach I’m prepared to deal with any sort of mild side effects. If it were to help, I’d be up for a bit of weight gain if that’s what it took.’*

(P42, female patient aged 18–25 years with severe IBS, 6-month interview)

#### Side effects and anticholinergic burden

GPs also expressed concerns about patient tolerability of side effects, potential of overdose, and wider impacts of prescribing amitriptyline, including anticholinergic burden in older patients:

*‘Often these patients are quite young, but as they get older, then it’s an anticholinergic and it adds to the anticholinergic burden and we know that people fall over and get confused and having long-term anticholinergic burden is not a great idea in terms of overall wellbeing.’*

(GP1, female GP aged 55–64 years with over 20 years’ experience)

#### Familiarity of amitriptyline

Despite concerns, the familiarity of amitriptyline appeared to help reassure patients and GPs about using amitriptyline for IBS. For patients, the well-established nature of amitriptyline as something that *‘has been tried and tested’* (P18) and *‘wasn’t like something that was brand new that I’d never heard of’* (P2), as well as previous personal experience or knowing others already taking amitriptyline for non-mental health conditions, appeared to reassure and normalise drug treatment, and specifically amitriptyline, for IBS:

*‘Well, I’ve got a couple of friends that are on them, but not for their tummies, they’ve got them because they’ve got a bit of stress in their lives, and have lost their husbands and stuff, and it helps them sleep. I was quite happy to go on them, because I knew that I’d got friends on it; it wasn’t something that I didn’t recognise.’*

(P9, female patient aged over 65 years with moderate IBS, 6-month interview)

Familiarity was also highlighted by GPs as potentially facilitating prescribing. GPs demonstrated existing knowledge of amitriptyline and seemed confident in making prescribing decisions about people with IBS. Amitriptyline was commonly viewed as a widely available and inexpensive treatment with well-established knowledge of side effects, which anecdotally *‘seems logical’* (GP11) that it could be successful in helping to resolve IBS symptoms:

*‘It’s freely available and, as I said, very cheap, so there isn’t going to be that barrier in terms of an expensive, new medication.’*

(GP2, male GP aged 35–45 years with 15–20 years’ experience)

Careful consideration of the suitability of amitriptyline for people with IBS was also evident. A common view was that prescribing amitriptyline to patients experiencing pain and diarrhoea, rather than constipation, was preferable due to a potential adverse effect of constipation and not wanting to *‘give them something that’s going to also constipate them’* (GP14).

#### Recognising potential benefits beyond IBS symptom relief

Patients and GPs highlighted potential benefits of amitriptyline other than treating IBS symptoms. A common misperception among patients was that taking low-dose amitriptyline might benefit their emotional health — *‘lift my mood a bit … lift my confidence a little bit’* (P12). Patients with previous experience of amitriptyline saw the potential to relieve symptoms in other pain-related conditions and improve sleep:

*‘I was crossing my fingers I was going to get amitriptyline because I had had it before, and it helped me sleep, and that was welcome. I think that was the naughty reason for wanting to do it. I mean, there was a good reason, but it’s also an additional motive for me if that makes sense.’*

(P40, male patient aged 45–54 years with moderate IBS, 6-month interview)

GPs also reflected on the utility of being able to *‘use some of the side effects to their advantage’* (GP8) and described how they promoted amitriptyline as a treatment with multiple benefits, particularly for addressing sleep problems:

*‘I think that there are going to be certain patients who, with their anxiety and their stress, they might have sleep problems. Having the option of using amitriptyline as an agent to help them with their IBS as well as their sleep is always, for some people in that sort of predicament, an attractive one.’*

(GP11, male GP aged 45–54 years with over 20 years’ experience)

### Meeting the desire for a novel approach to IBS

Patients’ desire for a cure and GPs wanting to offer more patient choice around IBS treatments seemed to contribute to an overarching theme that the potential positives of trying amitriptyline for IBS outweighed any concerns. Patients expressed frustration at having unresolved symptoms and feeling that they had tried everything else — *‘you name it; I think I’ve tried it; nothing seems to work’* (P30) — and, as such, were highly motivated to try something new, especially if it offered the hope of sustained symptom relief:

*‘That’s one of the reasons I was quite keen to come on this trial because it offered a ray of hope. A miracle cure! There are things you can try but none of them seem to be very effective. So really, if they could find those little blue pills that cure it, it would be brilliant.’*

(P1, male patient aged over 65 years with severe IBS, 6-month interview)

Although GPs did not view amitriptyline as a potential cure for IBS, they commonly expressed appreciation of having something else to offer patients, explaining that prescribing amitriptyline for IBS would allow them to add *‘another string to your bow’* (GP14) or have *‘another tool in the belt’* (GP3). GPs wanted reliable patient information about amitriptyline for IBS (like the information provided to patients in the trial) to help them offer it as an additional option for future patients:

*‘If there was a sort of information sheet about amitriptyline and IBS, so that would be quite … The side effects, how you can titrate it up yourself if it’s not working, and length of treatment and that sort of thing. Also maybe just revisiting the dietary and lifestyle things as well to complement that, “Don’t forget to manage your diet and manage your stress levels as well.” That would be quite helpful; that’s always a good thing.’*

(GP8, female GP aged 45–54 years with over 20 years’ experience)

## Discussion

### Summary

This qualitative study was nested within the ATLANTIS trial of low-dose amitriptyline for IBS, and 77 semi-structured interviews were conducted with patients and GPs. Thematic analysis identified concerns and perceived benefits from GPs’ and patients’ perspectives. These included concerns about possible stigma associated with a medication commonly viewed as an antidepressant, medicalising IBS and the associated health service burden, and the perceived potential for dependency and adverse effects. Perceived benefits included the familiarity of amitriptyline as a well-established drug, the low and flexible dose needed for IBS, and the potential for beneficial side effects (such as improved sleep). Concerns and perceived benefits were expressed in the context of a desire for a novel approach to IBS: GPs were keen to offer more options for IBS and patients sought a cure for their symptoms. This suggests that GPs and primary care patients with IBS may consider low-dose amitriptyline for IBS despite concerns, or after their concerns are appropriately addressed. Further thematic analysis then identified unique concepts, common concepts, and coherent threads that linked between GPs’ and patients’ perspectives, suggesting important implications for practice described below.

### Strengths and limitations

Despite using convenience sampling, a diverse sample of patients (in age, sex, education, and baseline symptoms, but not ethnicity) from each trial arm were interviewed, ensuring that we achieved a comprehensive account of patients’ perspectives. The lack of ethnic diversity reflects the participants recruited to the trial. Patients who have consented to take part in a placebo randomised trial and were willing to undertake the qualitative interviews may not hold the same views as people who did not participate. Unfortunately, no GPs from West Yorkshire had capacity to be interviewed because of the COVID-19 pandemic. Conducting interviews with the same patients at two time-points permitted the development of rapport and the evocation of experiences over a longer time period than is often achieved. This allowed us to see that patients’ views were broadly similar at 6 and 12 months. Future work might use diary methods or begin repeated interviews with patients earlier in their journeys with low-dose amitriptyline to further explore possible changes over time. Future trials might benefit from including a very early interview time-point (for example, at 2–4 weeks) to explore patients’ initial impressions and experiences of trial interventions. For ATLANTIS, this would have enabled us to capture patients’ perspectives on titrating closer in time to when they were engaged in this aspect of the trial. Interviewing patients and GPs permitted a more comprehensive analysis of views and experiences of low-dose amitriptyline for IBS than would have been possible by including only one of these groups. The multidisciplinary qualitative team, including input from patient collaborators, meant that we approached the data from diverse perspectives, achieving richer insights than might otherwise have been possible.

### Comparison with existing literature

Patients and GPs were encouraged to use amitriptyline for IBS by the low and flexible recommended dosage, its potential to offer benefits beyond IBS symptom relief including, for example, its effects on sleep, and its perceived ease of treatment (once-daily dosing and small tablets) including the patient self-titration process, which most participants found acceptable and empowering. Simple treatment regimens may facilitate adherence to medication,^31^ where adherence to complex dietary regimens such as a low FODMAP (Fermentable Oligosaccharides, Disaccharides, Monosaccharides And Polyols) diet can be poor^32^ and difficult for patients to manage in the context of daily life.^19^^,^^33^ Being able to reframe minor ‘adverse’ effects as potential benefits (such as effects on sleep) could help reduce patients’ concerns and thus might increase adherence.^34^^,^^35^ Empowering patients to self-titrate their dose, with the support of the dose titration document carefully developed for the ATLANTIS trial with PPI and clinician input, is consistent with increasing patient engagement^36^ and participation within patient-centred care.^37^ Other work has found using terms such as ‘neuromodulators’ rather than ‘antidepressants’ may be helpful, as well as explaining that in small doses the drugs target the ‘little brain in the gut’ as opposed to the big brain.^38^

Consistent with the common-sense model of illness perception^25^ and previous work in IBS,^19^^,^^39^ patient-perceived ongoing need for symptom relief facilitated patients’ uptake of a novel treatment, in this case low-dose amitriptyline for IBS. This is consistent with previous work examining the wider impact of IBS on patients’ lives and highlighting the challenges of treatment-seeking.^19^^,^^21^^,^^40^ That some patients expressed this in terms of wanting a ‘cure’ suggests that they may perceive IBS as an acute condition to be cured instead of a long-term condition to be managed.^41^ Consistent with the necessity-concerns framework,^34^ although patients expressed concerns about low-dose amitriptyline for IBS, these concerns were outweighed by the perceived need and desire for symptom relief and the benefits hoped for (at the start of the trial) and experienced (during the trial).

Our findings map to three key concepts from normalisation process theory,^42^ suggesting that low-dose amitriptyline for IBS made sense to GP interviewees (was ‘coherent’), that GPs appreciated its potential benefits for people with IBS and its ease of use, and therefore committed to it (‘cognitive participation’). To facilitate ‘collective action’, GPs valued additional patient-facing resources to support prescribing low-dose amitriptyline for IBS.

### Implications for research and practice

Future qualitative research that adopts more innovative approaches such as a real-time, longitudinal, or diary study would be beneficial and enhance the findings from this study. Future studies of uptake and adherence could also use questionnaires to relate patient perceptions quantitatively to patient behaviour. Such studies should note the salient concerns (such as about stigma) and benefits (for example, benefits of side effects) about low-dose amitriptyline for IBS identified by this study that are not measured by the leading generic questionnaire.^43^ Bespoke measures may be needed to fully capture patients’ beliefs about low-dose amitriptyline for IBS. Further research is needed to examine the potential anticholinergic burden risks of low-dose amitriptyline to inform prescribing.^44^^,^^45^

Low-dose amitriptyline for IBS is likely to be acceptable to, and often welcomed by, GPs and patients as an additional treatment option. The familiarity of amitriptyline may both hinder uptake (being associated with depression) and facilitate it (being a low dose of a medication taken by others with known mild side effects). Effective communication to address concerns and support informed patient-centred decision making should provide clear guidance about low-dose amitriptyline for IBS and anticholinergic burden, and resources for GPs and patients to distinguish low-dose amitriptyline for IBS from amitriptyline for other conditions (especially depression), and support patients managing their own dose-titration. Our PPI co-produced self-titration document and rationale for amitriptyline for IBS are currently being disseminated for use in clinical practice. They are freely available on the Leeds Clinical Trials Research Unit ATLANTIS trial website.^46^
