# Supplementary material for: Low-dose amitriptyline for irritable bowel syndrome: a qualitative study of patients’ and GPs’ views and experiences
Source: Br J Gen Pract. 2025 Mar 11;75(755):e431–9. doi: 10.3399/BJGP.2024.0303 (PMC11920899; doi:10.3399/BJGP.2024.0303)
Supplement: Supplementary file 1 [file BJGP.2024.0303_suppl.pdf]

<u>**Supplementary Materials**</u>

Supplementary Box S1: Atlantis Study Topic Guide: Patient Interviews

Iteration and Flexibility

The topic guide will evolve as the interviews progress in response to researchers' reflections, initial analysis, and feedback from participants. The scope of the interviews will always remain consistent with the objectives specified above. Within this scope, the topic guide will be used flexible in that the researcher may adjust the questions asked and the order in which they are asked as appropriate for the individual participant. It may not always be necessary to ask all of the questions in order to elicit the relevant information.

Introduction

1. Thank you for agreeing to take part in a research interview today. I am (*researcher name*), a researcher at University of Southampton. Just so you know, I am not a medical doctor so I'm afraid I can't offer any advice about your IBS today. [short hello]
2. I'm currently contacting a number of people who are taking part in the Atlantis study to find out about their experiences of the study, the study treatments, and their IBS.
3. How long this will take depends a bit on how much you want to tell us, but we anticipate it will take approximately one hour. Just let me know if you need to finish sooner and we can do that.
4. I would like to record the conversation we have today so that I can refer back to it at a later date. It enables me to listen to you better. We will store the recordings securely on a university password-protected computer. What we talk about will be used as part of the study, but what you say will remain anonymous. We're going to ensure this by not using your real name, or any other names that you mention, when we type up the interview. The typed up interviews will then be stored securely too. Is that ok?
5. Before we start there are a few things I'd just like to mention.
6. If I ask a question that you don't want to answer that is absolutely fine, just say so and I'll ask you a different question. If at any point you would like to take a break or stop altogether just tell me and we can do that.
7. Anything you can tell me about your experiences including good and bad points would be useful. What you say to me today will not impact your care in any way at all.
8. Do you have any questions before we start? Are you happy to continue?
9. **TURN ON THE RECORDING DEVICE**

Topic Guide

[Informed by the Common-Sense Model, CSM: illness perceptions, appraisals, beliefs about medicines]

<u>Opening (6m only)</u>

I'm really interested in finding out about how you came to participate in the study and what was like for you. Please could you tell me all about it?

<u>Initiating Atlantis (6m only)</u>

Thinking back to when you first heard about the Atlantis study, can you tell me what your IBS was like then?

Still thinking back to when you first heard about the Atlantis study, can you tell me anything about why you agreed to take part in it? Anything that made you want to be in it? Anything that concerned you about it? Had you ever been involved in medical research before?

What were your initial expectations and feelings?

What did other people think about you taking part in the study?

As you know, being in this study involved you taking some tablets. At the start of the study the researchers explained that some people would get amitriptyline and some people would get placebo pills. Can you tell me how you felt about this at the start of the study?

Did you have any thoughts or feelings about how taking amitriptyline might affect your IBS? [probe expectations of effect and concerns]

Did you have any thoughts or feelings about how taking a placebo might affect your IBS? [probe expectations of effect and concerns]

<u>Experiences during Atlantis (6m)</u>

How has your IBS been since you've been in the study? And your health/quality of life in general? [probe for anything getting better or worse]

Could you tell me all about your experience of taking part in the study so far?

Could you tell me about any consultations that you've had with your GP about your IBS since starting the study?

Could you tell me all about taking the tablets as part of the study? How was the process of dose adjustment? And talking to the research nurse on the phone?

There are lots of things that can mean we don't always take our medicines as prescribed. Was there anything that made it easier for you to take your tablets as prescribed? Was there anything that made it harder for you to take your tablets as prescribed? Have you had any concerns about the tablets? [prompt re side effects and probe re impact of these]

What did other people think about you taking the ATLANTIS tablets? [e.g. family, friends]

When you were taking the pills: Did you have any thoughts about whether they were amitriptyline or placebo? Did you think you received real pills or placebo pills? Why did you think so? What were the clues?

Is there anything else you would like to tell me about the pills that you were taking in the study?

Did you decide to continue or stop taking the tablets at 6 months? Can I ask how you came to that decision?

Have you started or stopped any other treatments for your IBS during the ATLANTIS study so far? [what and why] (prompt re prescribed and OTC meds and other treatments e.g. CBT/diet/complementary medicines)

<u>Experiences during Atlantis (12m)</u>

How has your IBS been since we last spoke? And your health/quality of life in general? [probe for anything getting better or worse]

Have you started or stopped any other treatments for your IBS since we last spoke? [what and why] (prompt re prescribed and OTC meds and other treatments eg CBT/diet/complementary medicines))

[For continuers only]: There are lots of things that can mean we don't always take our medicines as prescribed. Since we last spoke, was there anything that made it easier for you to take your tablets as prescribed? Was there anything that made it harder for you to take your tablets as prescribed? Have you had any concerns about the tablets?

When you were taking the pills: Did you have any thoughts about whether they were amitriptyline or placebo? Did you think you received real pills or placebo pills? Why did you think so? What were the clues?

Is there anything else you would like to tell me about the pills that you were taking in the study?

<u>Experiences of Study Procedures (6m+12m)</u>

How have you found being in the study, for example filling in the consent forms and the questionnaires, getting the medicines through the post, talking to the research nurse about the tablets? (prompt on each element of this question)

What, if anything, do you suggest researchers should do differently during the study process or to make it easier to take part?

Is there anything else you would like to say about taking part in the study? Do you think you would consider taking part in a study like this again in the future?

<u>Thoughts about the Future (6m+12m)</u>

Looking back on the treatment you've had in the study, what do you think about this treatment for IBS? Do you have any particular feelings about the use of amitriptyline for IBS?

Now that you've been in the study for (6/12m), what do you think will happen next with your IBS?

Is there anything that doctors could do differently to improve things for people with IBS?

And finally, is there anything else you would like to tell me about your experiences in the Atlantis study?

Supplementary Box S2: Atlantis Study Topic Guide: GP Interviews

Iteration and Flexibility

The topic guide will evolve as the interviews progress in response to researchers' reflections, initial analysis, and feedback from participants. The scope of the interviews will always remain consistent with the objectives specified above. Within this scope, the topic guide will be used flexible in that the researcher may adjust the questions asked and the order in which they are asked as appropriate for the individual participant. It may not always be necessary to ask all of the questions in order to elicit the relevant information.

Introduction

1. Thank you for agreeing to take part in a research interview today. I am (*researcher name*), a researcher at University of Southampton.
2. The aim of this interview today is to explore your experiences and views of amitriptyline for IBS and the Atlantis study.
3. With your agreement I will audio-record our conversation. The recording will be transcribed but everything you say will be anonymous. Your name along with any names you mention, any places you mention and all other identifiable information will be taken out, so that if someone read the transcript of your interview they would not know who you are or where you work. We will store the recordings and transcripts securely on a university password-protected computer.
4. Your interview will remain confidential.
5. If at any time you do not wish to answer a question that's okay, and if at any stage you wish me to stop the recorder, please let me know.
6. We can take a break at any time – let me know and I will stop the recording. We can either continue after the break, arrange another time to talk or stop there.
7. I would like to encourage you to be as honest as you can. There are no right or wrong answers. We are very interested in your views and experiences.
8. Do you want to ask me anything before we start? Are you happy to continue?
9. **TURN ON THE RECORDING DEVICE**

Topic Guide

[Informed by Normalization Process Theory, NPT: http://www.normalizationprocess.org/what-is-npt/npt-core-constructs/]

Starting the Atlantis Trial [NPT: Coherence]

When you first heard of the Atlantis study, what did you think about low dose amitriptyline for IBS?

How does offering amitriptyline for IBS differ from your usual practice for managing IBS? (prompt to get them to explain in detail their usual practice and any previous experiences of prescribing amitriptyline for IBS)

If you have previously prescribed amitriptyline for IBS – How did patients respond to a suggestion of trying amitriptyline for IBS? Prompt - did you encounter any resistance among patients to taking amitriptyline for IBS? What were patient's main concerns? How did you respond to that?

What is your sense of how the rest of your practice viewed amitriptyline for IBS prior to the ATLANTIS trial starting?

Interactions with Patients

Did you talk to any participants about amitriptyline or placebo for IBS during the ATLANTIS trial, e.g. at an optional GP review at 1 month, or at a routine appointment? [if no, do not continue this section] If yes how many?

How did patients invited to take part in ATLANTIS respond to a suggestion of trying amitriptyline or placebo for IBS? Prompt - Did you encounter any resistance among patients to taking amitriptyline or placebo for IBS? How did you response to that?

Did ATLANTIS participants report any difficulties taking the trial medication for IBS? How did you respond to that? Prompt – How did participants find the dose titration?

Reflections on Amitriptyline for IBS [NPT: Reflexive monitoring]

Now that you've completed work on the Atlantis study, what do you think about amitriptyline for IBS?

Did your thoughts change at all over the course of the trial, if so how?

What do you see as the benefits and drawbacks of amitriptyline for IBS? [For patients, for you, for your practice – probe on each element]

What is your sense of how the rest of your practice now view amitriptyline for IBS?

What changes did you have to make to your routine practice in order to offer amitriptyline for IBS in the Atlantis trial?

Are there any implications for your workload? Resource utilisation?

Amitriptyline for IBS in the Future [NPT: Reflexive monitoring]

Would you like to offer patients amitriptyline for IBS? Can you explain why?

What, if anything, would have to change for you to offer amitriptyline for IBS in routine practice? What could make this harder, what could make this easier?

Within the consultation, can you think of anything that might make it easier to prescribe amitriptyline for IBS? Anything that might make it more difficult? [Could be anything about you, the interaction, the patient, the setting, etc. etc.]

And what, if anything, would be the consequences of you offering amitriptyline for IBS in routine practice?
